# Synthesis of L-methionine-loaded chitosan nanoparticles for controlled release and their in vitro and in vivo evaluation

**DOI:** 10.1038/s41598-023-34448-6

**Published:** 2023-05-10

**Authors:** Pallath Muhammed Nuzaiba, Subodh Gupta, Shobha Gupta, Sanjay Balkrishna Jadhao

**Affiliations:** 1 Division of Fish Nutrition, Biochemistry and Physiology, Central Institute of Fisheries Education, Mumbai, Maharashtra 400061 India; 2 Department of Biotechnology, Annasaheb Vartak College of Arts, Commerce, Science, Vasai West, Mumbai, 401202 India

**Keywords:** Physiology, Nanoscience and technology

## Abstract

Therapeutically popular controlled release-enabling technology has forayed into the nutrition sector. Polymer coated forms of L-methionine used in soy protein diets, and its intermediate metabolite, *S*-adenosyl-L-methionine, used in myriad of medical conditions have proved more efficacious over (highly catabolized) free forms. In this premier study, L-methionine-loaded chitosan nanoparticles (M-NPs) were synthesized using ionic gelation method and their efficacy was evaluated. Biophysical characterization of the NPs was done using a Nanopartica SZ 100 analyser, transmission electron microscopy, and Fourier transform infrared spectroscopy. The M-NPs were spherical and smooth and 218.9 ± 7.4 nm in size and in vitro testing confirmed the controlled release of methionine. A 60-days feeding trial in *L. rohita* fish fingerlings was conducted. A basal diet suboptimal (0.85%) in methionine was provided with one of the supplements as under: none (control), 0.8% chitosan NPs (0.8% NPs), 1.2% L-methionine (1.2% M) (crystalline free form), 0.6% M-NPs and 1.2% M-NPs. While the addition of 0.6% M-NPs to the basal diet complemented towards meeting the established dietary requirement and resulted in significantly highest (*P* < 0.05) growth and protein efficiency and sero-immunological test scores (serum total protein, serum globulin, serum albumin: globulin ratio, phagocytic respiratory burst/NBT reduction and lysozyme activity), 1.2% supplementation in either form (free or nano), for being 0.85% excess, was counterproductive. Liver transaminases and dehydrogenases corroborated enhanced growth. It was inferred that part of the methionine requirement in nano form (M-NPs) can confer intended performance and health benefits in animals relying on plant proteins-based diets limiting in this essential amino acid. The study also paves the way for exploring chitosan NPs-based sustained delivery of amino acids in human medical conditions.

## Introduction

Animal-based and plant-based proteins meet the global demand for proteins. Animal proteins have superior amino acids profile, but their cost prohibits usage in farm species diets, despite preference, and are anathema to vegetarian people. Fishmeal is the most preferred protein in the diets of farm animals, especially fish and shrimp, in semi-intensive and intensive aquaculture systems that rely on a large quantity of aquafeed for optimal performance. While it is added to feed to stimulate intake in young fish and or to balance protein content, the currently stagnated fish meal production clearly predicts an unsustainable scenario, wherein, proteins from marine sources will no longer meet the demands of the aquafeed industry. Globally, there is a paradigm shift in feed production practices, which have already switched from marine to plant ingredients^1,2^ with the usage of feeding stimulants. The feed industries are extensively reliant on proteins from legumes and oilseeds. However, these plant proteins are poorly utilised due to the presence of anti-nutritional factors and often are not sufficient in one or other essential amino acids. For example, despite the *numero uno* position of soybean meal for being the preferred and predominantly used source of plant protein in fish feeds, it is limiting in methionine (cells use only L*-*forms). Methionine is known to enhance feed intake and promote growth in fish^3^ and acts as a methyl donor for DNA methylation^4–6^. Deficiency of methionine, from exclusive or extensive use of soymeal (i.e., no or low fish meal) in diets is corrected by supplementation of a synthetic source of L-methionine.

Across species, the use of free crystalline amino acids helps to make up dietary deficits and devise optimum environmental friendly diets^7,8^, but poses limitations as they are absorbed and catabolised faster due to temporal mismatch in availability at the sites protein synthesis^9^. In addition, they are prone to leaching in water when used in aquatic feed and are potentially degraded in the rumen in herbivores. As such, dietary crystalline methionine in its free form is not very efficient^10^ and is economically expensive and requires 10% more than the NRC requirement to achieve growth in fish^11^. Secondly, increasing the amount of methionine in the diet may not be an alternative or good option, because the excess amount could turn toxic^12^ and increase the feed cost. Further, a low dose, long-term supplementation of free amino acids is better utilised than short-term high dose^13^, but can compromise dietary levels. Thus, the best strategy that emerges is the use of slow-release systems for free amino acids. While Guo et al.^14^ opined that supplementing methionine in coated or microform would improve its bioavailability, Yúfera et al.^15^ reported a 68% reduction in the leaching of free amino acids (FAA) into the water in 1 h with the use of the microencapsulated diet over a gelatin microbound diet.

In humans, *S*-adenosyl-L-methionine (SAMe) therapy has been suggested and positive results have been obtained in diseases such as depression, dementia, liver diseases (cholestasis, steatohepatitis, cirrhosis), pancreatitis, vacuolar myelopathy, osteoarthritis and type II diabetes^16–18^. Because only 1% of the administered dose reaches the circulation due to its degradation in the gastrointestinal tract, transit in the liver and low permeability, the recommended dose of 800–1600 mg a day for a variety of clinical conditions cannot be met. While SAMe is relatively expensive and unstable, its precursor, methionine can be a good choice for supplementation. However, there are only a few studies on improving its bioavailability. Controlled release systems over coated amino acids are also desired for people with inborn errors in amino acid metabolism e.g. phenylketonuria^19–21^.

Cellulose-acetate-phthalate, acrylic-resin, tripalmitin-polyvinyl alcohol^22^, or natural materials like alginate, carrageenan, agar, gum arabic and zein have been used for coating amino acids to improve bioavailability, but reports on nanocomplexes and their in vivo evaluation are scarce. The unique size-dependent property of nanoparticles (NPs) enables the delivery of virtually any small molecule for targeted disease and health outcomes. Classified by the FDA as generally regarded as safe (GRAS), biodegradable polymers are used as a promising carrier for the delivery of biomolecules and drugs^23,24^. Chitosan, a natural biopolymer, is considered to be the most suitable and sustainable nanocarrier of pharmaceutically and nutritionally active compounds for oral delivery because of its non-toxic, non-irritant nature^25^, biocompatibility and mucoadhesive property^26–29^. It shields the load from the adverse conditions of the gastrointestinal tract^30^. Chitosan is cationic in nature which makes it soluble across wide pH gradients in acidic and basic solutions. The addition of polyanionic compounds in chitosan solution under specific conditions would lead to the formation of NPs by ionic gelation or poly-electrolyte complexing^31^. Encapsulation of therapeutic drugs in chitosan NPs is known to not only protect them from leaching and physiological barriers of the gut^32^ but also enable their controlled release and absorption^33^ and cellular uptake^34,35^. We have synthesized chitosan nanoparticles and used them for delivery of proteolytic enzymes^36^ and RNA^37^ orally and hormone^38^ through injection with beneficial effects in fish. There are only a few studies on improving its bioavailability. Earlier, Ergin, et al.^39^ synthesized and evaluated i*n vitro* chitosan NP-based systems for colon targeted delivery of *S*-adenosyl-L-methionine. In this pioneering study, methionine-loaded chitosan NPs were fabricated and tested in vitro for methionine release and in vivo for their ability to promote growth performance and immune profile of a model fish, *L. rohita,* which is one of the major cultivable species in freshwater aquaculture.

## Materials and methods

### Chemicals

Low molecular weight chitosan (MW < 200 kDa) with a deacetylation degree of 90% and the sodium salt of tripolyphosphate were purchased from Sigma-Aldrich (Saint Louis, MI, USA). Purified feed ingredients were procured from Hi-Media (Hi-Media Laboratories, Mumbai, India). Other chemicals and consumables used in the study were of analytical grade.

### Preparation of chitosan nanoparticles

Chitosan nanoparticles were prepared by ionic gelation method^31^. Five different ratios of chitosan to L-methionine (1:0.5, 1:1, 1:1.5, 1:2, and 1:2.5) were employed to make L-methionine-loaded chitosan NPs. 0.2 g of purified chitosan flakes were dissolved in 10 ml of 10% acetic acid. A pH of 5.74 was reached by adding 0.2 M sodium hydroxide solution, and the total volume was made up to 100 ml bringing the final concentration of chitosan to 0.2%. The solution was sterile filtered using a 0.22 µm syringe filter. Sodium tripolyphosphate (TPP) (0.7 mg ml^−1^) solution was then added dropwise to the chitosan solution to allow the formation of nanoparticles. Loading of L-Methionine (referred to without rotational conformation hereafter) was done by dissolving it in the chitosan solution before adding TPP. The nanoparticles were freeze-dried for experimental diet preparation.

### Characterisation of nanoparticles

#### Measurement of particle size

The hydrodynamic mean diameter of the nanoparticles and their size distribution was determined by a dynamic light scattering (DLS)- nanoparticle size analyser, Nanopartica SZ-100 (Horiba Ltd, Kyoto, Japan). All measurements were performed in a wavelength of 633 nm at 25 °C with an angle detection of 90°.

#### Determination of zeta potential

The zeta potential measurements were made using Nanopartica SZ-100 (Horiba Ltd, Kyoto, Japan) by applying the principle of Laser Doppler Electrophoresis. Lyophilised samples were diluted in deionised water and determined in triplicates.

#### Transmission electron microscopy (TEM)

Transmission Electron Microscopy (TEM) (FEI Tecnai G2 F30) at Sophisticated Analytical Instruments Facility (SAIF), Indian Institute of Technology (IIT), Mumbai, was used to observe the morphology of chitosan nanoparticles. The lyophilised nanoparticles were suspended in deionised water to 1/100 (v/v) and stained with 2% phosphotungstic acid (a negative stain) 1 h before observation.

#### FT-IR spectroscopy

A Fourier transform infrared spectrophotometer (FT-IR, Bruker, Germany) of the Sophisticated Analytical Instrument Facility (SAIF) at the Indian Institute of Technology (IIT), Mumbai, was used to investigate the interaction of functional groups between methionine and chitosan. The lyophilised nanoparticles were taken with KBr pellets and pressed to a plate for this reason.

#### Determination of encapsulation efficiency

The optimum loading capacity for methionine during the ionic gelation procedure was determined by calculating the encapsulation efficiency of chitosan nanoparticles- loaded with methionine in ratios of 1:0.5, 1:1, 1:1.5, 1:2 and 1:2.5. The encapsulation efficiency (EE) of the chitosan NPs was determined as the percentage ratio of the encapsulated methionine to the total methionine according to the equation; EE% = Encapsulated methionine/Total methionine × 100. The amount of the encapsulated methionine was indirectly determined as the difference between the total amount of methionine initially added and that of the free and unencapsulated methionine. For this, free methionine in the aqueous medium was separated from nano-encapsulated methionine by ultracentrifugation at 40000×*g* at 4 °C for 45 min and the free methionine in the supernatant was measured by the nitroprusside method^40^. Briefly, 1 ml of 5N NaOH and 0.5 ml of 1% sodium nitroprusside were added to 1 ml of the supernatant. Samples were subjected to photometric measurements at 520 nm in a UV–VIS spectrophotometer (Shimadzu UV 1601 spectrophotometer, Tokyo, Japan) after 10 min of incubation at room temperature.

### In vitro release study

In vitro release of methionine was tested on nanoparticles formulated using a 1:1.5 chitosan to methionine ratio for their highest encapsulation efficiency (83.63 ± 1.67%) among combinations tried. The release rate of methionine from the chitosan NPs was measured in phosphate- buffered solution (pH 7.4) at 37 °C in triplicate. Briefly, 10 mg of freeze-dried nanoparticles having the desired load of methionine were taken in screw-capped tubes and dispersed in a 10 mL phosphate-buffered saline (PBS) solution. The tubes were placed in a shaking water bath (Julabo, SW3; Seelbach, Germany) maintained at 37 °C and shaken horizontally at 90 rpm. The tubes were taken out of the water bath at predetermined time intervals and centrifuged at 40000×*g* for 30 min at 25 °C. The supernatant was carefully collected and replaced with an equal volume of fresh buffer solution. The quantity of released methionine in the supernatant was analysed by the nitroprusside method.

### Fish and feeding trial

Fingerlings of *Labeo rohita* species were brought from the local fish farm and acclimatized in a circular tank (2000 L) for 15-days and fed a diet with 30% crude protein. The feeding experiment was conducted in the wet laboratory for 60-days in FRP tanks having 150 L capacity. The tanks were arranged as per a completely randomised design and there were three for each treatment and four replicates for the control group. Fifteen acclimatized fingerlings of *L. rohita* (mean weight 2.8 ± 0.41 g) were stocked in each tank. The experimental feeding was carried out twice a day at 9:00 a.m. and 4:00 p.m. up to satiation level and uneaten feed was siphoned out. All fish from each experimental tank were weighed every 15 days to assess the biomass and adjust feeding rate calculation during the trial period. During the study period, the water quality parameters were in the optimal range required for the *L. rohita* fingerlings and are presented in Table S1. The research conducted was approved by the statutory authorities of the ICAR-Central Institute of Fisheries Education, Mumbai 400,061. Guidelines of the Committee for the Purpose of Control and Supervision of Experiments on Animals (CPCSEA, Ministry of Environment & Forests, Animal Welfare Division, Government of India) were followed. The provisions of the Wildlife Protection Act of 1972 are not applicable for experiments on *L. rohita* as it is not an endangered fish.

### Experimental diets

The basal control diet containing about 31.2% crude protein and 3530 kcal/kg digestible energy (DE) was formulated using purified ingredients (Table 1). To make experimental diets, an equal quantity of a non-nutritive ingredient, cellulose, was replaced by the item that defined treatment (0.8% chitosan NPs, 1.2% free L-methionine, 0.6% M-NPs and 1.2% M-NPs. All the ingredients were weighed accurately as per the requirements based on their percent content in the diet. The first six ingredients (see Table 1) were appropriately mixed to make a dough and steam cooked for 30 min. The rest of the ingredients along with methionine, chitosan nanoparticles, or L-methionine-loaded chitosan nanoparticles were sprayed into the steam -cooked dough after cooling. Nanoparticles used in the experimental diet were synthesized using chitosan to methionine in the ratio of 1:1.5. The amount of methionine in the nanoparticles used for diet preparation was ensured by calculating the encapsulation efficiency. The composition of all five diets used in the study is given in Table 1. Two levels (0.6 and 1.2%) of sustained release nano-methionine were selected for supplementation of the basal diet that contained 0.85% indigenous protein-bound methionine estimated following standard methods, McCarthy and Sullivan^40^ and Kader et al.^41^. One level was set close to safely meeting the reported methionine requirement of 1.15% to 1.2%^42–44^ for carps on a similar type of diet. The dough was pressed through a pelletizer to get uniform-sized pellets, which were spread on a sheet and air-dried. After drying, the pellets were packed in cryo bags and stored in the deep freezer.

**Table 1:** Ingredient composition and sulphur amino acid content (%) of the experimental diets.

Ingredients	Control	0.8% NPs	1.2% M	0.6% M-NPs	1.2% M-NPs
Casein	33.00	33.00	33.00	33.00	33.00
Gelatin	8.25	8.25	8.25	8.25	8.25
Dextrin	16.75	16.75	16.75	16.75	16.75
Starch soluble	20.38	20.38	20.38	20.38	20.38
Cellulose	10.07	9.27	8.87	8.67	7.91
CMC	1.50	1.50	1.50	1.50	1.50
Cod liver oil	4.00	4.00	4.00	4.00	4.00
Sunflower oil	4.00	4.00	4.00	4.00	4.00
Ascorbic acid	0.03	0.03	0.03	0.03	0.03
Vit.-Min. Premix^a^	2.00	2.00	2.00	2.00	2.00
BHT	0.02	0.02	0.02	0.02	0.02
Chitosan NPs (free)		0.80		0.32	
L-methionine			1.20		
M-NPs complex^b^					
Methionine				0.60	1.20
Chitosan NPs				0.48	0.96
Total	100.00	100.00	100.00	100.00	100.00
Total methionine (%)	0.85	0.85	2.05	1.45^c^	2.05^d^
Cystine (%)	0.12	0.12	0.12	0.12	0.12
Proximate analysis^e^ and other LAA^f^					

*CMC* carboxymethyl cellulose, *BHT* butylated hydroxytoluene.

^a^Composition of the standard premix in our lab is described in detail earlier^47^.

^b^M-NPs-L-Methionine loaded Chitosan NPs (Chitosan to methionine ratio of 1:1.5).

^c, d ^Of the total percent content, 0.6% and 1.2% (nano) methionine is released slowly.

^e^All the feeds were isonitrogenous and isocaloric and contained on DM basis: crude protein 31.19 ± 2.6, ether extract 8.83 ± 0.07, total CHO (TC) 51.03 ± 0.40*,* ash 8.14 ± 0.29, and digestible energy (DE) 353.10 ± 3.14 kcal.

DE (Kcal/100 g) = (% CP × 4) + (% EE × 9) + (TC × 4)*.* (n = 3), (As per AOAC method).

^f^Other limiting amino acid contents: lysine: 2.66%, tryptophan: 0.019% and arginine: 2.02%.

### Amino acid composition of experimental diets

The method of Kader et al. was followed. In brief, a 100 mg dry sample was weighed and placed into clean dry hydrolysis glass tubes (Thermo Fisher 29,571), which was then filled with 1 mL of 6N hydrochloric acid. After that, all of the air was evacuated from the tubes using a vacuum pump, and the tubes were filled with nitrogen before sealing tightly. The sealed hydrolyzing tubes were then heated for 24 h on a dry block heater at 110 °C. The tubes were then removed from the block heater and allowed to cool to room temperature. The 10 mL cooled digested sample was then transferred to a 15 mL centrifuge tube with the help of a 3 mL syringe fitted with a long stainless-steel needle, and the hydrolyzing tube was cleaned by washing out the particulate matter with 9 ml of BioChrom loading buffer. The pH of a 10 mL sample was then adjusted to between 2 and 2.2 using 7.5 M NaOH solution and filtered through a 25 mm syringe filter. Then, a 200 µL filtered sample was placed into a 1.5 mL sample tube along with 800 µL BioChrom loading buffer. This tube was then placed in the autosampler, and the amino acid concentrations of samples were determined using an automated amino acid analyzer (BioChrom 30 +, Biochrom Ltd, United Kingdom).

### Sampling and analytical methods

At the end of the 60-days feeding trial, five fish from each tank were collected and anaesthetised with clove oil (50 ml/L) for the collection of blood samples. While quantification of respiratory burst activity/ nitroblue tetrazolium (NBT) reduction^45^ and blood glucose (Somogyi method, see^46^) was done immediately on some samples, serum was separated from the rest for estimation of lysozyme activity^47^, total serum myeloperoxidase content^48^ and serum complement-3 (C_3_) (ELISA kit) (CUSABIO, Houston, TX USA), serum total protein and albumin (Qualigens Diagnostics, Mumbai, India). Serum globulin was calculated by subtracting albumin from total serum protein. Fish were sacrificed and muscle and liver tissues from the same fish were aseptically dissected out and kept in an appropriate buffer with 0.25% sucrose as cryoprotectant and stored in a deep freezer. Tissue samples were homogenised using phosphate buffer (pH 7) containing 0.25% sucrose and 0.1 X Halt™ protease inhibitor and the supernatant was collected after centrifuging at 7000×*g*. Activities of aspartate aminotransaminase (AST) (E.C. 2.6.1.1) and alanine aminotransaminase (ALT) (E.C. 2.6.1.2), lactate dehydrogenase (LDH; L-lactate NAD^+^ oxidoreductase: E.C. 1.1.1.27) malate dehydrogenase (MDH; L-malate: NAD^+^ oxidoreductase: EC.1.1.1.37) were measured as described earlier^45,49^. Myotomal muscle glycogen content was determined following KOH (30%) digestion of weighed sample at 100 °C for 15 min. Ethanol was used to precipitate glycogen from an aliquot of the digest^50^, and the precipitated glycogen was assayed by the anthrone method^51^.

### Data collection and growth efficiency study

Growth performance of fingerlings was evaluated in terms of weight gain (%), specific growth rate (SGR), food conversion ratio (FCR), and protein efficiency ratio (PER) based on the following standard formulae: Weight gain (%) = (Final weight − initial weight)/initial weight × 100; SGR (% day^−1^) = (Log_e_ final weight − Log_e_ initial weight)/number of culture days × 100; FCR = total dry feed intake(g)/wet weight gain(g) and PER = total weight gain(g)/crude protein fed (g).

### Statistical analysis

Tanks (with 15 fish in each) constituted an experimental unit for data on growth, while pooled samples (e.g. serum) from fishes were a unit for the rest of the data such as biochemical and immuno-hematological measurements. The data were analysed using the IBM Statistical Package, SPSS, version 22.0 (SPSS Inc., Chicago, IL). The data tested for normality (Shapiro–Wilk’s test) and homogeneity of variance (Levene’s test) were subjected to one-way ANOVA. The significant levels of difference for all measured parameters were calculated and means were compared by Duncan’s Multiple Range Test at a 5% probability level. The *p* value < 0.05 was considered statistically significant.

## Results

### Synthesized L-methionine-loaded chitosan nanoparticles have desired physical characteristics

**Figure 1:**
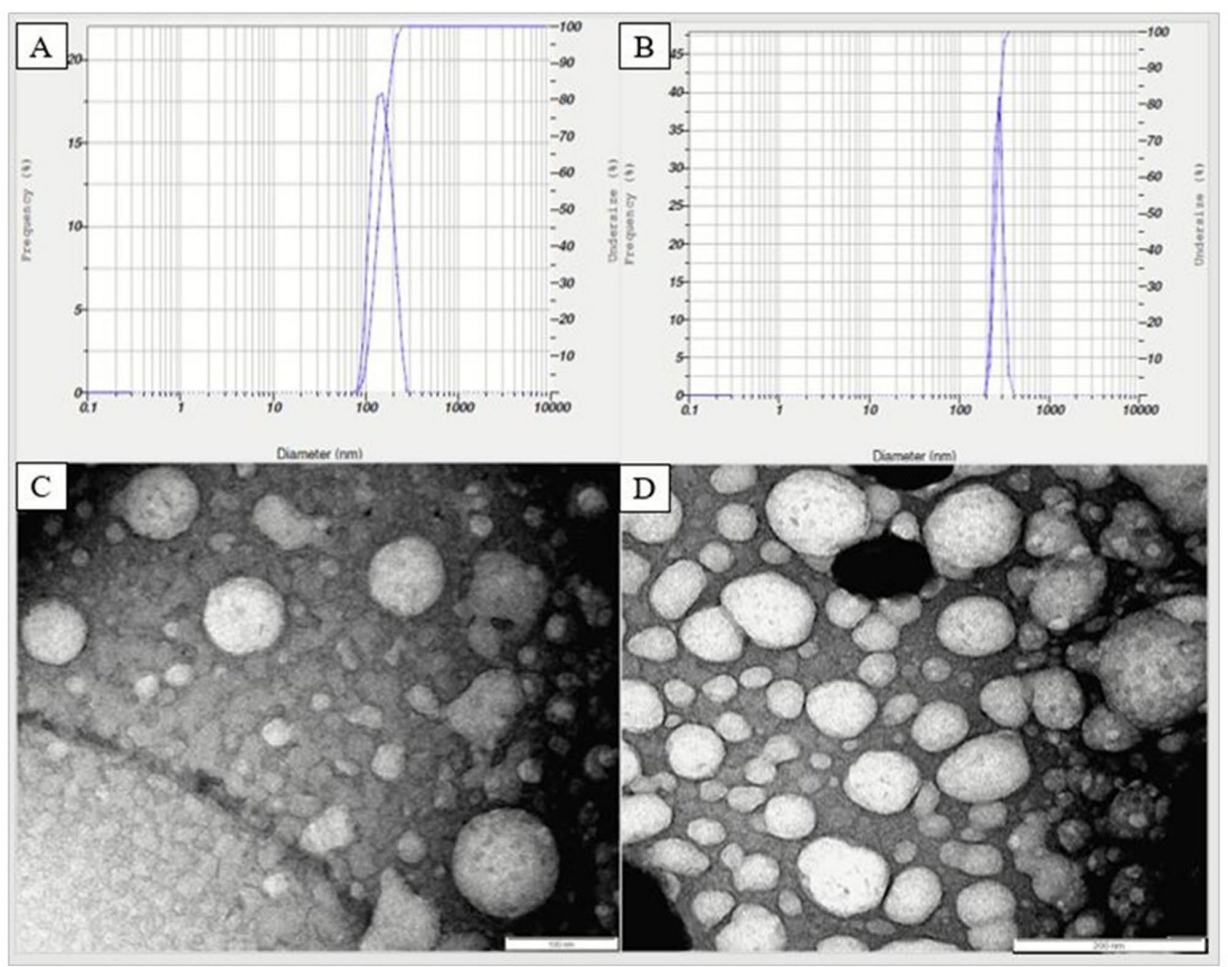
Particle size distribution. (**A**) Chitosan nanoparticles; (**B**) Methionine encapsulated chitosan nanoparticles; (**C**) Transmission electron microscopic images of the non-encapsulated form of chitosan nanoparticles; and (**D**) Chitosan nanoparticles after encapsulation of methionine.

A comparison of the physical and chemical characteristics of the synthesized chitosan and methionine-loaded chitosan nanoparticles (NPs) is given in Table 2. During the process of making M-NPs, as the proportion of methionine increased from 0.5 to 2.5 in fixed 1 part of chitosan NPs, the size of chitosan M-NPs kept on increasing (*P* = 0.000), but zeta potential (*P* = 0.036) and encapsulation efficiency (EE%) (*P* = 0.000) increased until methionine proportion was 1.5, where optimum loading capacity was found. However, zeta potential and encapsulation efficiency were decreased when the methionine proportion was highest (NPs: Met ratio, 1:2.5). Following encapsulation of methionine in chitosan NPs the polydispersity index was reduced from 0.72 to 0.52 (Fig. 1A,B). Transmission electron spectroscopy (TEM) images of methionine-loaded chitosan nanoparticles (Fig. 1C,D) exhibited almost spherical and dispersed particles in the medium.

**Table 2:** Characteristics of methionine-loaded chitosan nanoparticles.

Chitosan TPP NP: methionine ratio	Size (nm)	Zeta potential	Encapsulation efficiency
Chitosan (NPs) blank (1:0)	173.26^a^ ± 12.1	28.6^a^ ± 3.7	–
NPs: Met (1:0.5)	180.09^a^ ± 3.67	27.77^a^ ± 1.23	51.78^a^ ± 1.92
NPs: Met (1:1.0)	208.9^b^ ± 1.53	25.33^a^ ± 1.76	65.86^c^ ± 1.4
NPs: Met (1:1.5)	218.9^bc^ ± 7.4	31.37^b^ ± 1.6	83.63^e^ ± 1.67
NPs: Met (1:2.0)	232.07^cd^ ± 4.79	28.33^ab^ ± 1.45	74.69^d^ ± 2.06
NPs: Met (1:2.5)	252.33^d^ ± 14.13	25.33^a^ ± 1.76	57.78^b^ ± 1.83

Chitosan NPs: Methionine ratio was varied by using fixed chitosan nanoparticle concentration and different methionine concentration (0, 0.4, 0.8, 1.2, 1.6 and 2%).

Values are represented as mean ± standard error (n = 3).

^a,b,c,d,e^Means bearing different superscript letters in the same column differs significantly (*P* < 0.05).

### FT-IR spectra confirm L-methionine loading onto chitosan NPs

**Figure 2:**
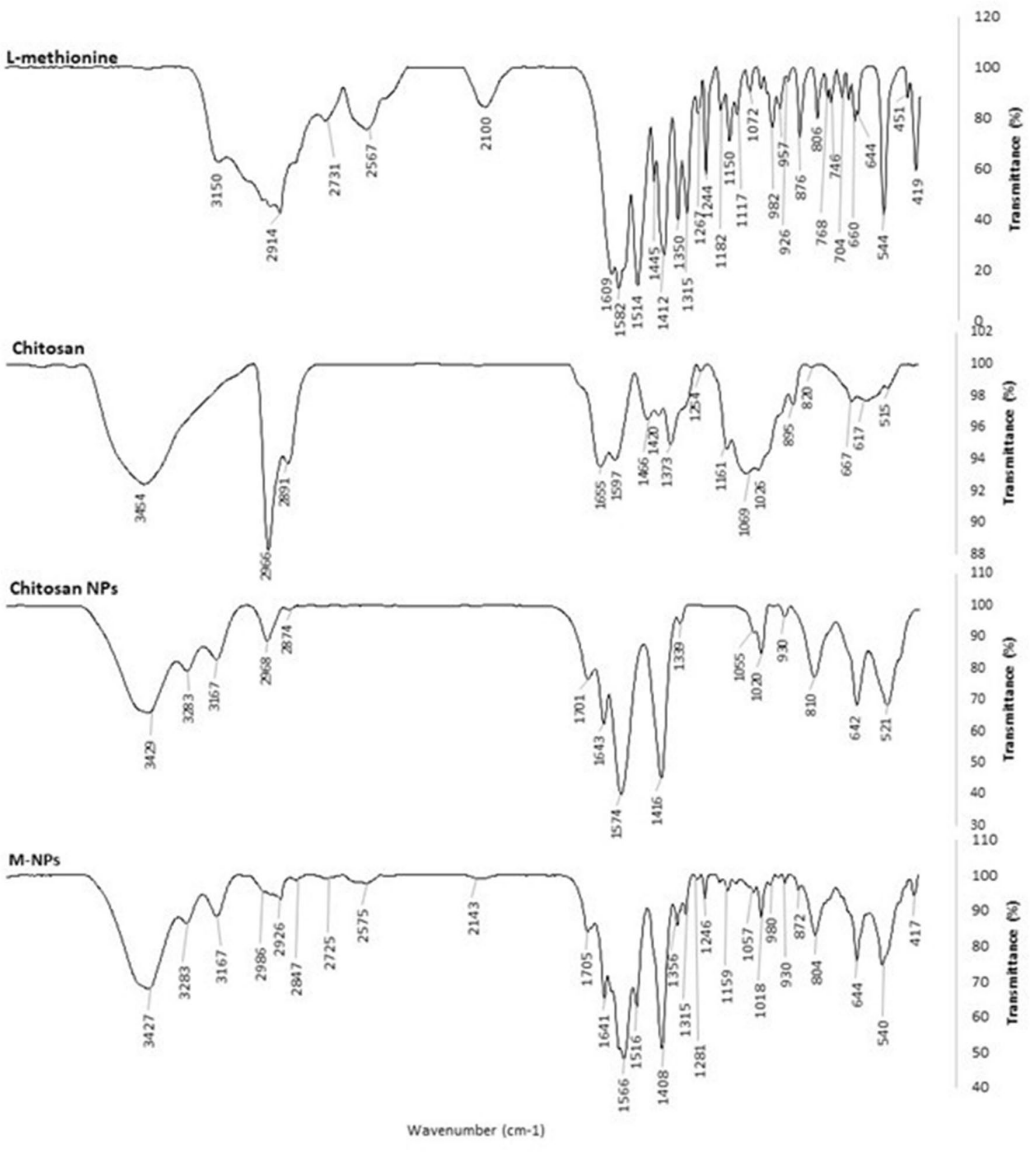
FT-IR spectra of L-methionine, chitosan, chitosan nanoparticle (NPs) and L-methionine loaded chitosan nanoparticle (M-NPs).

Spectral analysis using FT-IR revealed potential interactions of L-methionine with chitosan nanoparticles. The spectral broadband wavelength for L-methionine, chitosan, chitosan NPs and L-methionine-loaded chitosan NPs (M-NPs) ranged from 3500 to 3300 cm^−1^ (See Fig. 2). A peak at 3454 cm^−1^ in chitosan was shifted to 3427 cm^−1^ in nanoparticulate forms. The peak of a broad band at 3150 cm^−1^ in the L-methionine spectrum disappeared and was not visible in M-NPs. The peak at 2914 cm^−1^ in L-methionine and 2968 in chitosan NPs was shifted to 2926 cm^−1^ in M-NPs. A small peak at 2600–2550 cm^−1^ was found only in L-methionine and L-methionine-loaded nanoparticles spectra. The peak at 1609 cm^−1^ in L-methionine and at 1643 cm^−1^ in chitosan NPs was found at 1641 in M-NPs. The peak at 1514 cm^−1^ in L-methionine is reflected in M-NPs as well. A peak found in L-methionine at 746 cm^−1^ disappeared in M-NPs. Peaks at 644, 660 and 764 cm^−1^ were visible in both L-methionine and M-NPs.

### *Slow and controlled release of L-methionine from chitosan nanoparticle *in vitro

**Figure 3:**
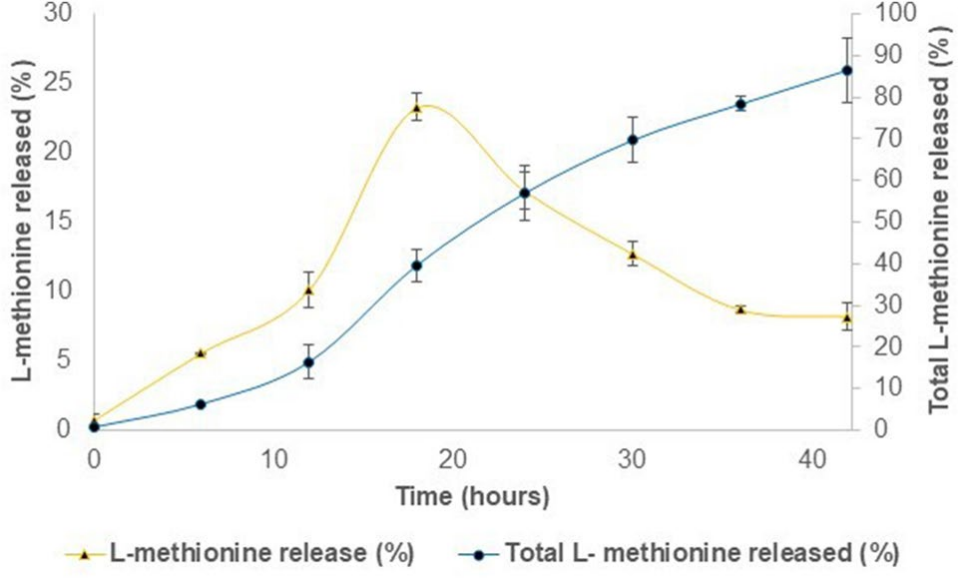
In vitro release profiles of L-methionine from chitosan nanoparticles. Chitosan to methionine ratio used for the study was 1:1.5. The proportion of L-methionine released to the initial L-methionine load is indicated in the secondary axis as total L-methionine released (%.) Proportion of L-methionine released to the Methionine content after each sampling is L-methionine release (%). Results were reported as mean ± standard error, n = 3.

The nanoparticles having desired characteristics showed a consistently slow-release rate with only 10% release until 12 h of incubation, which increased faster in the next 6 h reaching a peak (23%) at 18 h (Fig. 3) and then slowly decreasing thereafter until 42 h. Of the loaded amount, 16%, 40%, and 86.5% total methionine was released at the end of 12, 18, and 42 h, respectively.

### Superior performance in fish when nano-methionine partly covers the total methionine requirement

**Figure 4:**
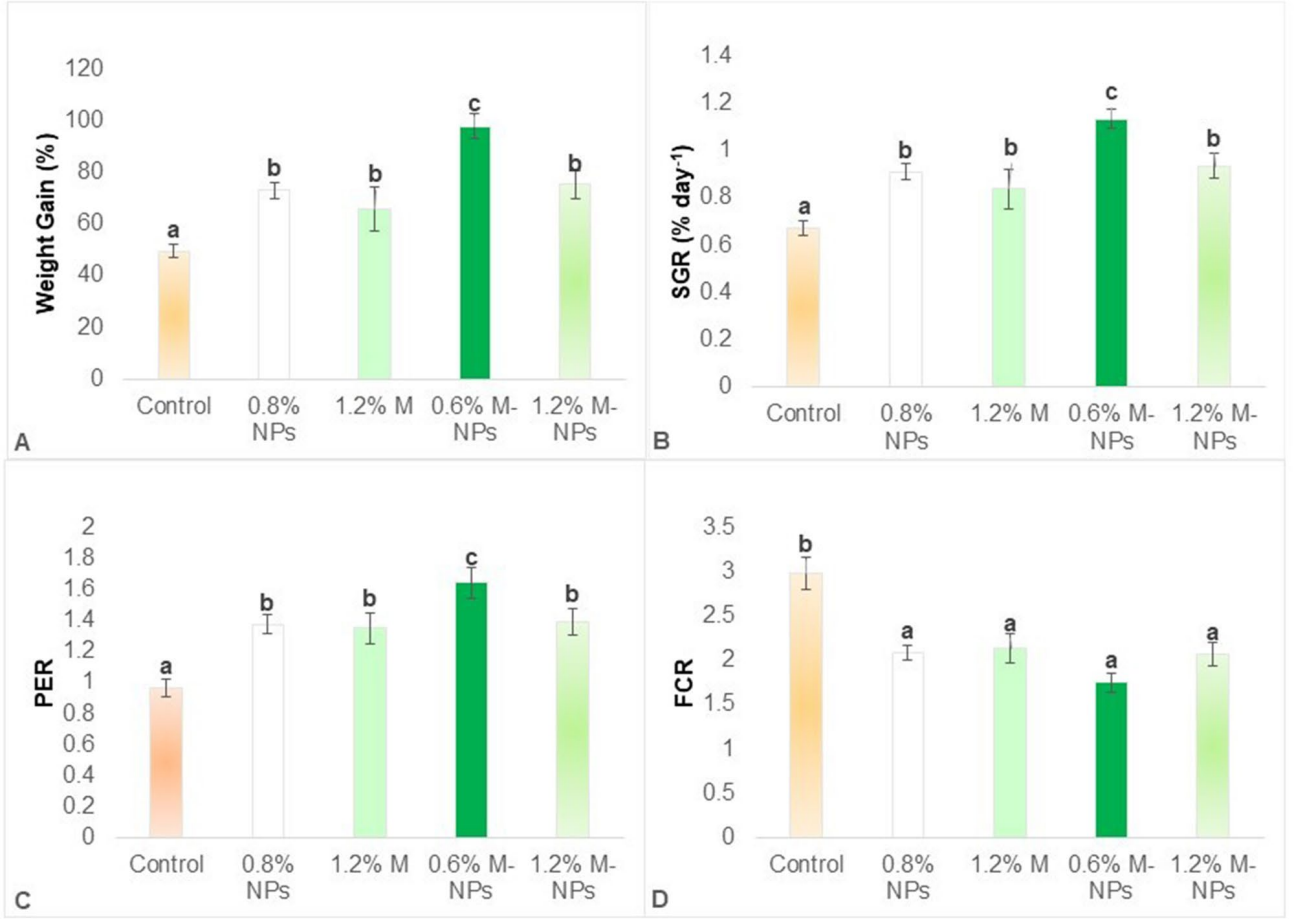
Growth and performance of *L. rohita* fish fed diets containing methionine-loaded in chitosan nanoparticles for 60 days. (**A**) Weight Gain (%); (**B**) SGR: Specific Growth Rate; (**C**) Protein Efficiency Ratio (PER); and (**D**) Food Conversion Ratio (FCR). Abbreviations: *0.8% NPs* 0.8% chitosan nanoparticles; *1.2% M* methionine in free form (M); *0.6% M-NPs or 1.2% M-NPs* methionine loaded in chitosan nanoparticles (M-NPs). Values are presented as means ± standard error (control, n = 4; treatments, n = 3). ^a,b,c^Means bearing different superscript letters differ significantly (*P* < 0.05).

**Figure 5:**
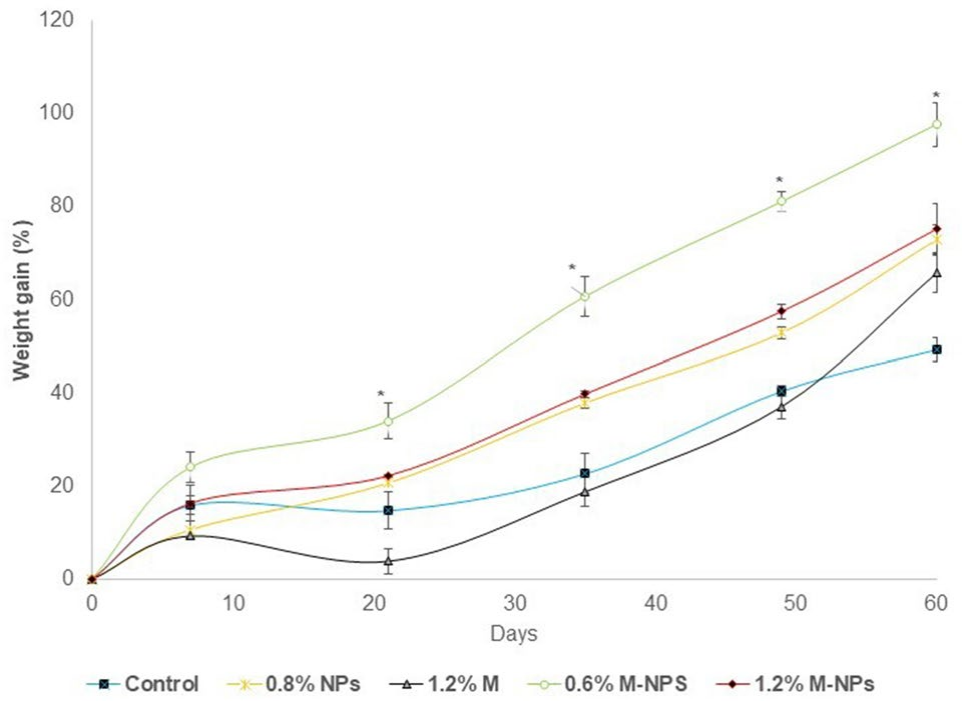
Time- interval *g*rowth pattern of *L. rohita* fish fed diets containing methionine loaded in chitosan nanoparticles for 60 days. Abbreviations: *0.8% NPs* 0.8% chitosan nanoparticles; *1.2% M* methionine in free form (M); *0.6% M-NPs or 1.2% M-NPs* methionine loaded in chitosan nanoparticles(M-NPs). Values represented as means ± standard error (control, n = 4; treatments, n = 3). *Mean significantly (*P* < 0.05) different from the rest of means on the respective day.

Growth and performance responses of *L. rohita* fish given different dietary treatments are depicted in Fig. 4A–D. Compared to the control, the addition of chitosan NPs, 0.6% M-NPs and 1.2% methionine in free (1.2%M) or nano form (1.2%M-NPs) to the basal diet improved performance parameters such as percent weight gain (%WG), specific growth rate (SGR), feed conversion ratio (FCR) and protein efficiency ratio (PER) (P = 0.000 for %WG and SGR and *P* = 0.001 for FCR and PER). While chitosan NPs improved (*P* < 0.05) % WG, SGR and PER of fish, the effect was significantly potentiated (*P* < 0.05) on 0.6% methionine-loaded chitosan NPs (0.6% M-NPs), which together with casein-bound methionine helped fulfil the required dietary concentration, but the performance of 1.2% M-NPs was alike methionine in free form (1.2% M), the level that contributed 0.85% excess methionine. From the third week to the eighth week, the differences in per cent weight gain between 0.6% M-NPs supplemented methionine adequate and 1.2% M (free form) and 1.2% M-NPs supplemented methionine excess diet were significant and ranged to the tune of 30 to 40% and 10 to 20%, respectively (Fig. 5).

### Metabolic responses corresponded to growth and performance in fish

**Figure 6:**
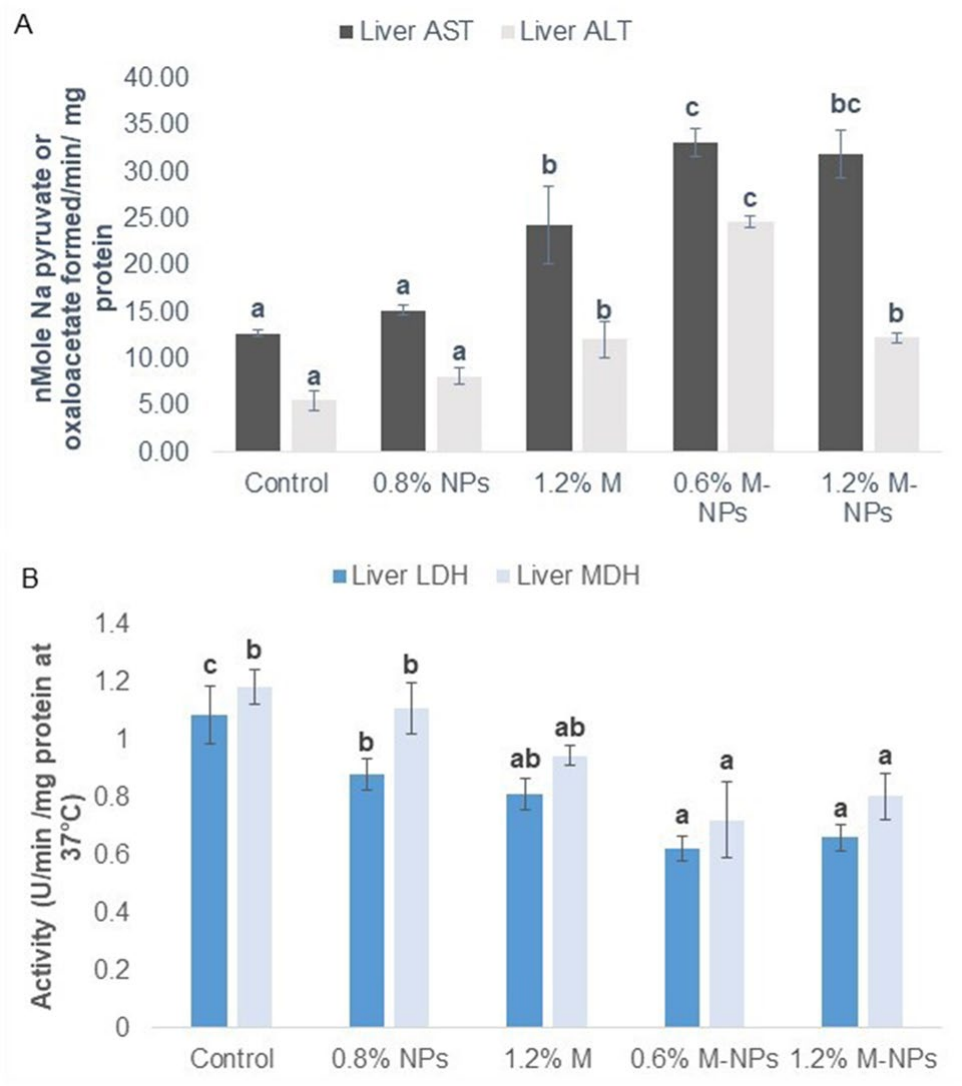
The activity of liver transaminases (**A**) and dehydrogenases (**B**) of *L. rohita* fish fed diets containing methionine loaded in chitosan nanoparticles for 60 days. Abbreviations: *0.8% NPs* 0.8% chitosan nanoparticles; *1.2% M* methionine in free form (M); *0.6% M-NPs or 1.2% M-NPs* methionine loaded in chitosan nanoparticles (M-NPs). Values represented as means ± standard error (ALT and AST, n = 3 to 5; LDH and MDH, n = 3). ^a,b,c^Means bearing different superscript letters differ significantly (*P* < 0.05).

There were significant differences in hepatic AST (*P* = 0.000), ALT (*P* = 0.000), LDH (*P* = 0.003), and MDH (*P* = 0.015) in response to NPs. Liver ALT and AST activities of the *L*. *rohita* fingerlings were not influenced by plain chitosan NPs (*P* > 0.05). While activities of both transaminases in nano (0.6% M-NPs) supplemented-methionine-adequate diet were significantly higher than that of the free form 1.2% M diet, ALT activity in 1.2% methionine via either form (free or nano) was similar and AST in the 1.2% M-NPs group did not differ from that of the 0.6%M-NPs (Fig. 6A). Compared to the control, feeding 0.8% chitosan NPs and 1.2% methionine (1.2 M) decreased (*P* < 0.05) liver LDH. A comparison of liver dehydrogenases, LDH, and MDH activity, in both 0.6% and 1.2% M-NPs was found similar to the 1.2% M methionine-fed group (Fig. 6 B). The blood glucose level (*P* = 0.098) and muscle glycogen (*P* = 0.268) were similar among treatment groups (Table 3).

**Table 3:** Blood glucose and muscle glycogen of *L. rohita* fish fed diets containing methionine loaded chitosan nanoparticles for 60 days.

Treatments	Blood glucose (mg dl^−1^)	Muscle glycogen (mg/g)
Control	91.89 ± 3.87	1.41 ± 0.06
0.8% NPs	82.55 ± 4.84	1.55 ± 0.12
1.2% M	88.21 ± 2.07	1.37 ± 0.09
0.6% M-NPs	101.29 ± 9.93	1.54 ± 0.20
1.2% M-NPs	92.07 ± 5.97	1.45 ± 0.04

*0.8% NPs* 0.8% chitosan nanoparticles; *1.2% M* methionine in free form(M); *0.6% M-NPs or 1.2% M-NPs* methionine loaded chitosan nanoparticles (M-NPs).

Values represented as means ± standard error (n = 3).

### Improved sero-immunological profile in fish fed methionine-loaded chitosan nanoparticles

**Figure 7:**
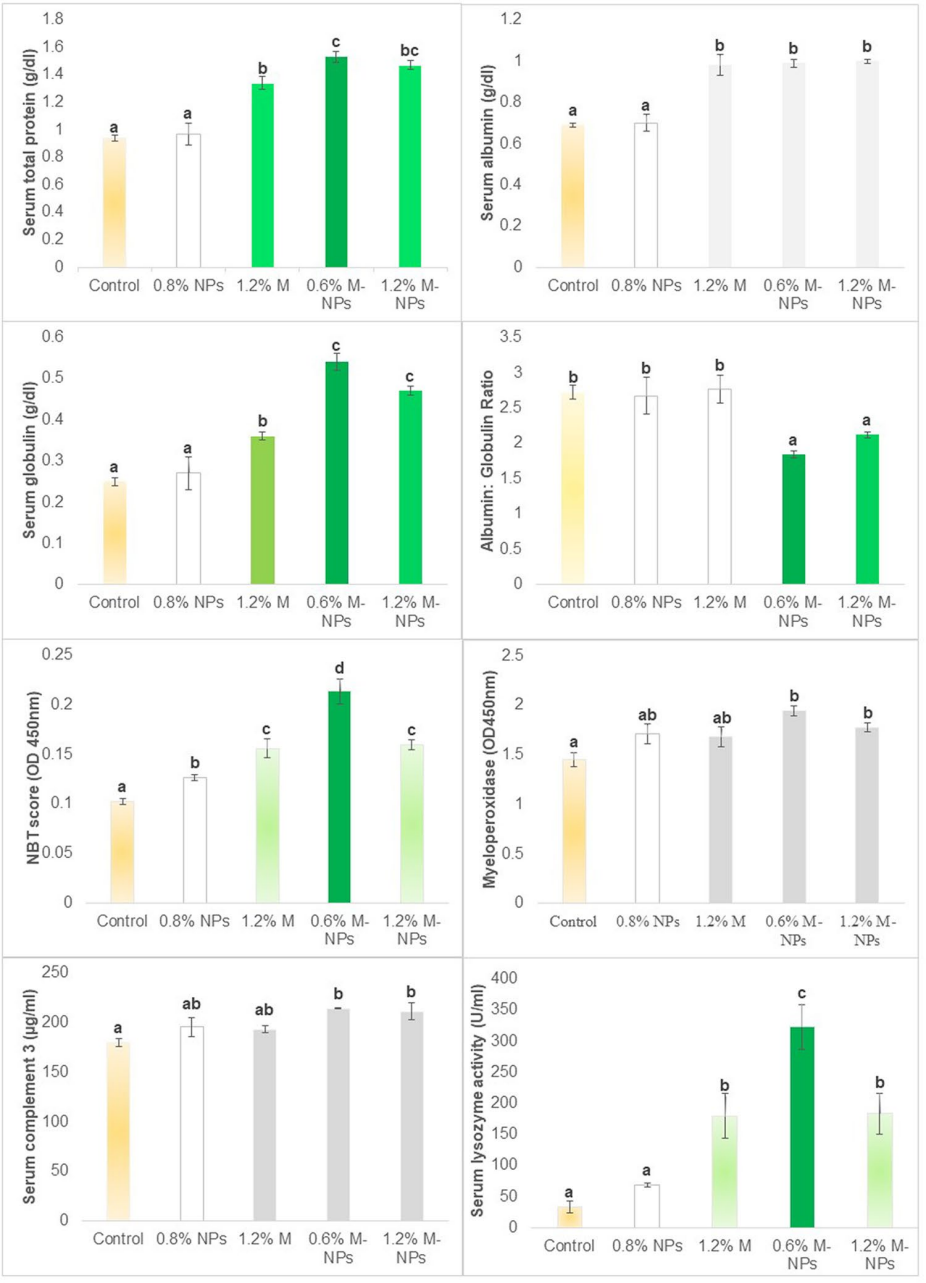
Immunological parameters of *L. rohita* fish fed diets containing methionine loaded chitosan nanoparticles for 60 days. Abbreviations: *0.8% NPs* 0.8% chitosan nanoparticles (NPs), *1.2% M* methionine in free form (M); *0.6% M-NPs or 1.2% M-NPs* methionine loaded chitosan nanoparticles (M-NPs). Values represented as means ± standard error (n = 6 for NBT and 3 for the rest). ^a,b,c^Means bearing different superscript letters differ significantly (*P* < 0.05).

Dietary NPs had significant effects on fish’s serological attributes like total serum protein, albumin, and globulin (all *P* = 0.000) and immunological indicators such as albumin: globulin (A:G) ratio (*P* = 0.005), NBT score/respiratory burst (*P* = 0.001), myeloperoxidase (*P* = 0.018), serum complement, C3 (*P* = 0.018) and lysozyme (*P* = 0.002) (Fig. 7). Unaffected by chitosan NPs, serum total protein, albumin, and globulin were increased by feeding 1.2% M, but only total protein and globulin were further potentiated (*P* < 0.05) by feeding it in nanoform at 0.6% level (0.6% M-NPs), with 1.2% (1.2% M-NPs) retaining the effect on only globulin, while the form of methionine had no bearing on serum albumin of fish. As a result, the A:G ratio in fish fed on nanoform of methionine at both levels was improved (*P* < 0.05).

Significantly highest (*P* < 0.05) respiratory burst activity and lysozyme activity was found on 0.6% M-NPs, a level that complemented the attainment of the required dietary concentration, as against 1.2% free (1.2% M) or nano form (1.2% M-NPs), which contributed 0.7% excess methionine and yielded lower scores in these tests. Compared to the basal diet subnormal for methionine, all five tests (respiratory burst, lysozyme, MPO activity, serum complement 3, and serum A: G) showed improved immune profile on nano-methionine-fed diets. Compared to the control, supplementation of 0.8% chitosan NPs increased (*P* < 0.05) respiratory burst activity in fish.

## Discussion

Chitosan nanoparticle-based delivery system never tried before have been attempted in order to address various issues associated with methionine nutrition in aquatic species. Methionine and cysteine are sulphur containing amino acids, which are needed for the synthesis of sulphur-containing biochemicals. As the only variable in diets was methionine, cysteine (and other amino acids) being constant, only the former is the focus of discussion.

Chitosan NPs synthesised by ionic gelation method have similar size distribution^31^. Size of the NPs was slightly increased after loading methionine, which agrees with earlier reports^52–54^. The hydrated form of NPs before (173 nm) and after (218 nm) loading was 1.28 to 2.47 times larger than that of dehydrated nanoparticles (70–150 nm) (TEM images). The larger size of hydrated NPs is due to the extended and large size conformation of chitosan in aqueous solution^55^, and swelling or aggregation of single particles dispersed in water^53,56^. TEM images showed the spherical and smooth surfaces of chitosan NPs. Similarly, chitosan NPs^57^ without or with vitamin C^28^ or bovine serum albumin (BSA)-loading^55^ were found to exhibit a smooth spherical shape.

Chitosan has mucoadhesive characteristics due to the positive charge of protonated amine groups on it at pH less than 6.2^26,28^. The NPs before and after loading of L-methionine had a surface charge density (zeta potential) around + 30 mV, which is the minimum for physically stable NPs in an aqueous solution^58,59^. The positive surface charge helps to exclude NPs from lysosomal reduction^60^ hence they could be targeted to the cytoplasm^61^.

Good encapsulation of methionine in chitosan NPs needs defined conditions. When chitosan and tripolyphosphate (TPP) are mixed, it forms a compact nano-complex by cross-linking the positively charged amide group on chitosan and the negatively charged phosphate group on TPP^59^. Loading of L-methionine on chitosan NPs at pH 6.0 (the isoelectric pH of L-methionine, pI is 5.7 and pI of chitosan is 6.2) was found to be most suitable. BSA- loaded chitosan NPs were in a range of 250–350 nm with 61–78%^62^ encapsulation efficiency. Relatively, high encapsulation in the present study is attributed to the hydrophobic interactions formed between chitosan and hydrophobic amino acids^63^.

Fourier transforms infrared (FT-IR) spectroscopy further ensured the encapsulation of L-methionine in chitosan NPs. A broad peak at 3500–3300 cm^−1^ is attributed to overlapped stretches of carboxyl O–H, N–H and aliphatic C-H bonds. A weak band between 3300 and 2950 cm^−1^ is attributed to C–H stretching vibrations in L-methionine. The peak shifting from 3454 cm^−1^ in chitosan NPs to 3427 cm^−1^ in L-methionine-loaded chitosan NPs is attributed to N–H and O–H stretching vibrations^64^. Similarly, the peak at 3150 cm^-1^ corresponding to N–H stretching vibrations of a primary amine in L-methionine^65^ disappeared after the formation of M-NPs. The peak at 2914 cm^−1^ in L-methionine and 2968 cm^−1^ in chitosan were attributed to CH_2_ stretching vibrations^57^, which shifted to 2926 cm^−1^ in M-NPs. The band from 2600 to 2550 cm^−1^ attributed to weak S–H stretching of the thiol group was found only in L-methionine, and L-methionine-loaded NPs spectra. Wave number assigned to asymmetric stretching of CH_3_-S of L-methionine at 746 cm^−1^ disappeared in L-methionine-loaded NPs, while highly variable CH_2_-S stretches of L-methionine at 644, 660 and 764 cm^−1^ were visible in L-methionine-loaded NPs as well^66^. All these findings further ensured that chitosan NPs could be used to encapsulate L-methionine.

In vitro test results of 40% methionine release in 18 h, or 86.5% release at the end of 42 h shows a slow, delayed, and sustained release of methionine from chitosan NPs. In one study^39^, 51.29% *S*-adenosyl-L-methionine (SAMe)-loaded onto nano-chitosan was released at the end of the first hour, while 70% of pure SAMe was dissolved. Using the same vehicle, chitosan NPs, 96% SAMe release in about 14 h^67^, and only 12% ciprofloxacin release in 96 h^68^ have also been reported. The release of methionine might occur by diffusion, which involves penetration of medium into the system, causing swelling and then release^33,52,69^. A drag observed in the earlier hours of the in vitro release test may indicate the time taken up for swelling of chitosan NPs in the medium. Such slow release of drug from NPs is also attributed to the decomposition of the carrier^69^. There is a caveat to bear in mind while extending results on the release of methionine at pH 7.4 at 37 °C during in vitro tests to the actual gut of animals. In a more realistic release kinetics study^70^, the chitosan nanoparticles system ensures a burst delivery efficiency of 50% in 6 h (and 70% in 12 h) of rifampicin at a simulated intestinal pH in comparison to the simulated gastric environment of less than 10%.

Chitosan NPs are well-proven active carriers of drugs used for effective oral delivery^71^, and hence supplementation of methionine by loading in chitosan nanoparticles enhanced the growth of fish in the current investigation. The positive charge of chitosan NPs interacts with the negatively charged epithelial membrane and induces structural reorganisation to enhance transport through the paracellular pathway^72,73^. It is also reported that the bi-adhesion nature of chitosan NPs helps in controlled release^26,28^ and its mucoadhesive property can extend the residence time of NPs in the gastrointestinal tract^73^. In the present study, the base level of 0.8% chitosan was ensured while preparing M-NPs to negate their effect on the parameters studied. By doing so, the significantly highest growth rate was found in the 0.6% M-NPs supplemented diet, where M-NPs contributed to gross methionine adequacy of diet through methionine’s slow release from chitosan NPs and its efficient absorption in the fish lumen.

Although reports on amino acids/M-NPs are not available, a few on coated forms of methionine or lysine showed encouraging results. When 0.3% acrylic resin coated methionine supplemented diet containing 46% CP was fed, 23.64% higher weight gain and 7.99% higher specific growth rate of the juvenile cobia, *Rachycentron canadum* (Linnaeus) was found^22^. Successful replacement of 21.2% fish meal by a mixture of protein with 0.5% lysine and (hydrogenated palm oil) coated 0.34% methionine in the diet of black sea bream, *Acanthopagrus schlegelii* has been attempted^74^. Compared to an optimal methionine diet containing 0.6% M-NPs, supplementation of 1.2% methionine in either form (nano or free) to the basal diet caused 0.7% methionine excess leading to decreased % weight gain, specific growth rate and protein efficiency ratio. A significant decrease in these parameters was also observed in another Indian major carp, *Cirrhinus mrigala*^43^ and in channel catfish, *Ictalurus punctatus*^75^ when fed 0.5 to 0.75% more methionine than requirement.

Increased fish growth on controlled release form 0.6 M-NPs can be attributed to the availability of methionine in plasma for extended hours. Studies in rats on a single oral dose of free *vs* chitosan-encapsulated chlorogenic acid (CGA), a phenolic antioxidant, clearly indicated that the level for free CGA peaks at 1.5 h and then reaches baseline at the end of 2 h, while for encapsulated CGA, plasma concentration reaches the peak at 4 h and then comes to baseline at 6 h. Such lower and sustained release over a longer duration (*P* < 0.01) of nano-CGA has more bioavailability upon oral administration^76^ which is also likely for methionine. Rolland et al.^9^ reported that supplementation with crystalline amino acids in a plant protein-based diet resulted in the rapid appearance of amino acids in the plasma, especially a greater and faster rise of methionine, followed by sharp decreases compared to fish meal. Such highest plasma availability of amino acids results in a temporal mismatch at the site of protein synthesis in rainbow trout (*Oncorhynchus mykiss* W.), leading to a decrease in growth and an increase in ammonia excretion^77^, which can be avoided with the usage of slow releasing nano-methionine, M-NPs, at an appropriate level, as used in this study. Studies^78^ in grass carp, *Ctenopharyngodon idella,* suggest significantly (*P* < 0.05) increased growth coupled with high activity of intestinal enzymes such as trypsin, lipase and amylase, Na+ /K+ -ATPase, alkaline phosphatase (AKP), *γ*-glutamyl transpeptidase (*γ*-GT) and creatine kinase (CK) (in one of the segments) in fish given methionine close to their dietary requirement, while decreased (*P* < 0.05) growth and compromised activities were noticed when dietary methionine level was 1.5 to 2 times higher than the requirement. This explains the good growth patterns on 0.6% M-NPs but not 1.2% in M or 1.2%M-NPs. Although, chitosan at 2 to 5% is known to display growth-enhancing effect in a variety of fish species^79–81^, this effect on 0.8% level of chitosan nanoparticles in this study suggests more effectiveness of chitosan NPs over plain chitosan.

Since amino acids are primary sources of energy in fish^82^, enzymes involved in their metabolism were studied. High energy demand for protein synthesis in fish is achieved by increasing the gluconeogeic pathway. Substrate for this pathway is provided by transaminases, especially AST and ALT^82^. Activities of these enzymes in the liver corresponded with energy-demanding protein synthesis processes such as growth and immuno-synthesis, which were highest in 0.6% M-NPs than 1.2% methionine (in free or nano form) indicating increased utilisation of dietary protein towards growth. Direct correlation between growth and transaminases activity has been shown in *L. rohita*^36^ and in *Rachycentron canadum*^83^. Our results agrees with Wu, et al.^78^ who reported decreased growth in grass carp (*Ctenopharyngodon idella*) along with decreased hepatopancreatic AST, but not ALT activity on either side (0.5 × less or 2 times more) of the required dietary level. Similarly, reduced lactate and malate dehydrogenase activity corresponds to increased growth in fish, which is attributed to reduced glycolytic breakdown during growth^49,84^. In this study, both LDH and MDH were comparatively lower in M-NPs fed groups than in control, but not in 1.2% free methionine-fed fish. Further, blood glucose and muscle glycogen levels showed no significant difference between treatment groups, which indicates the effective utilisation of glucose produced via gluconeogenesis^36^.

Immuno-serological tests employed in the study for fish fed with M-NPs are robust and well-validated tests. Macrophage exhibits respiratory burst activity for destroying invading pathogenic microbes, which can be determined by nitroblue-tetrazolium (NBT) reduction assay^85^. Myeloperoxidase enzyme uses H_2_O_2_ to produce HCl during the respiratory burst. Lysozymes acts as an opsonin which activates phagocytes and the complement system^80^. The activated complement system destructs foreign organisms by opsonizing phagocytes^86^. Further higher serum protein content, globulin content and low A:G ratio indicate the immune boosting activity of an immunostimulant^87,88^.

Methyl donors are well known immunomodulatory agents and our studies on betaine and choline, compounds involved in one-carbon metabolism (methionine cycle), have convincingly demonstrated these properties in fish^45,47^. Significantly improved scores of three (respiratory burst activity of neutrophils, lysozyme activity and serum albumin: globulin ratio) of five (myeloperoxidase and serum complement 3) immune profile parameters were found in the diet in which 0.6% nano-methionine (0.6% M-NPs) contributed to methionine adequacy of the basal diet. On the other hand, higher than required methionine through supplementation of 1.2% in either form (free or nano) to basal diet with 0.85% methionine in intact protein was of no use as fish were not challenged, a situation which may increase its requirement. In yellow catfish, *Pelteobagrus fulvidraco*^89^, dietary methionine close to requirement (0.97% and 1.17%) showed the highest respiratory burst activity, lysozyme and total globulin than fed with subnormal (lower) or higher levels up to 1.64%. Increased complement (C3) activity and lysozyme activity were found at 1–1.2% of free L-methionine supplied groups in juvenile Jian carp^44,86^ and in European seabass^90^. Similarly, Wu et al.^78^ convincingly demonstrated the importance of a normal level of methionine in the diet of sub-adult grass carp, *Ctenopharyngodon idella,* for the maintenance of levels of numerous antioxidant indicators such as malondialdehyde, protein carbonyl content, catalase, glutathione-S-transferase, anti-superoxide anion, anti-hydroxyl radical, superoxide dismutase, glutathione peroxidase, glutathione reductase and glutathione contents in hepatopancreas and intestine. In fish fed on either side of the requirement level (~ 0.62%) (0.5 × times less or 1.5 to 2 times higher) of this amino acid, the first four attributes were significantly increased, and the rest were decreased (*P* < 0.1) or remained unaffected and unchanged. The present study harnessed immune boosting^79,91,92^ and metabolism modulating- properties of chitosan^79,81^ to develop immune response in fish^93^. Chitosan NPs synergistically and effectively acted with L-methionine towards improving immune profile in addition to growth and performance when given as potent M-NPs at a level not to exceed the total (intact and free) dietary methionine level.

## Conclusion

This is the pioneering report on the synthesis of L-methionine-loaded chitosan nanoparticles (M-NPs). These M-NPs during in vitro testing exhibited sustained and slow release of methionine over a prolonged period. Complementing diets limiting in methionine with M-NPs (nano methionine) to meet its dietary requirement was found to confer intended performance and health benefits in fish. Specific amino acid-loaded chitosan nanoparticles such as M-NPs in this study can find dietary applications to achieve desired goals in animal production and health practice, where a slow and controlled release of this amino acid is desired.

## Supplementary Information

Supplementary Information.

## Data Availability

All data generated or analysed during this study are included in this published article.
