# Supplementary material for: Synthesis of L-methionine-loaded chitosan nanoparticles for controlled release and their in vitro and in vivo evaluation
Source: Sci Rep. 2023 May 10;13:7606. doi: 10.1038/s41598-023-34448-6 (PMC10172396; doi:10.1038/s41598-023-34448-6)
Supplement: Supplementary file 1 — Supplementary Information. [file 41598_2023_34448_MOESM1_ESM.docx]

| Dissolved Oxygen | 5.8–7.3 mg.L^-1^ |
| --- | --- |
| Temperature | 23.6–27.5 °C |
| pH: | 7–8.1 |
| Free Carbon Dioxide: | Negligible |
| Total hardness | 228–245 mg L^-1^ |
| Ammonia-N | 0.16–0.25 mg L^‑1^ |
| Nitrite-N | 0.002–0.004 mg L^‑1^ |
| Nitrate-N | 0.02–0.06 mg L^‑1^ |

**Supplementary File**

**Table S1**. Quality parameter of the water used for rearing the *L. rohita* fingerlings.
